# Poly[[μ_10_-4,4′-(ethane-1,2-diyldi­oxy)dibenzoato]dipotassium]

**DOI:** 10.1107/S1600536812008513

**Published:** 2012-03-03

**Authors:** Zhen Ma, Baohuan Liang, Wanbing Lu

**Affiliations:** a Guangxi Key Laboratory of Petrochemical Resources, Processing and Process Intensification Technology, School of Chemistry and Chemical Engineering, Guangxi University, Nanning, Guangxi 530004, People’s Republic of China

## Abstract

The title salt, [K_2_(C_16_H_12_O_6_)]_*n*_, was obtained by the reaction of 1,2-bis­[4-(ethyl-carbox­yl)-phenox­yl]ethane with KOH in water. The anion lies on a crystallographic inversion center, which is located at the mid-point of the central C—C bond. The K^+^ cation is coordinated by six O atoms, two from the chelating carboxyl­ate group of the anion and four from four neighboring and monodentately binding anions, giving rise to an irregular [KO_6_] coordination polyhedron. The coordination mode of the cation leads to the formation of K/O layers parallel to (100). These layers are linked by the nearly coplanar anions (r.m.s. deviation of 0.064 Å of the carboxyl, aryl and O—CH_2_ groups from the least-squares plane) into a three-dimentional network.

## Related literature

For the preparation, structures, properties and applications of metal carboxyl­ate compounds, see: Ma *et al.* (2005 ▶); Su *et al.* (2010 ▶); Zhang & Chen (2008 ▶); Zhu *et al.* (2008 ▶). For the preparation of the precusor, see: Ma & Yang (2011 ▶). For standard bond lengths, see: Allen *et al.* (1987 ▶).
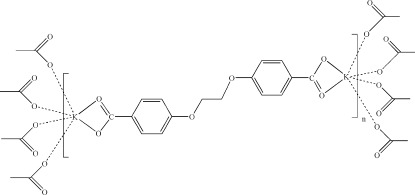


## Experimental

### 

#### Crystal data

[K_2_(C_16_H_12_O_6_)]
*M* *_r_* = 189.23
Monoclinic, 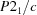

*a* = 18.0696 (7) Å
*b* = 3.9866 (1) Å
*c* = 11.3189 (5) Å
β = 107.188 (2)°
*V* = 778.96 (5) Å^3^
*Z* = 4
Mo *K*α radiation
μ = 0.64 mm^−1^
*T* = 298 K
0.15 × 0.11 × 0.10 mm

#### Data collection

Bruker SMART CCD area-detector diffractometer
Absorption correction: multi-scan (*SADABS*; Sheldrick, 1996 ▶) *T*_min_ = 0.919, *T*_max_ = 0.938
7387 measured reflections
2146 independent reflections
1999 reflections with *I* > 2σ(*I*)
*R*_int_ = 0.018

#### Refinement

*R*[*F* ^2^ > 2σ(*F* ^2^)] = 0.023
*wR*(*F* ^2^) = 0.067
*S* = 1.01
2146 reflections
109 parameters
H-atom parameters constrained
Δρ_max_ = 0.39 e Å^−3^
Δρ_min_ = −0.22 e Å^−3^

### 

Data collection: *SMART* (Bruker, 2001 ▶); cell refinement: *SAINT* (Bruker, 2002 ▶); data reduction: *SAINT*; program(s) used to solve structure: *SHELXS97* (Sheldrick, 2008 ▶); program(s) used to refine structure: *SHELXL97* (Sheldrick, 2008 ▶); molecular graphics: *XP* in *SHELXTL* (Sheldrick, 2008 ▶); software used to prepare material for publication: *publCIF* (Westrip, 2010 ▶).

## Supplementary Material

Crystal structure: contains datablock(s) I, global. DOI: 10.1107/S1600536812008513/wm2592sup1.cif

Structure factors: contains datablock(s) I. DOI: 10.1107/S1600536812008513/wm2592Isup2.hkl

Additional supplementary materials:  crystallographic information; 3D view; checkCIF report

## Figures and Tables

**Table 1:** Selected bond lengths (Å)

K1—O2^i^	2.6558 (8)
K1—O1^ii^	2.6780 (8)
K1—O2	2.7069 (8)
K1—O1^iii^	2.7167 (8)
K1—O2^iv^	2.8027 (8)
K1—O1	3.0335 (8)

Symmetry codes: (i) 
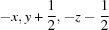
; (ii) 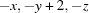; (iii) 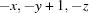; (iv) 
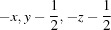
.

**Fig. 1.**
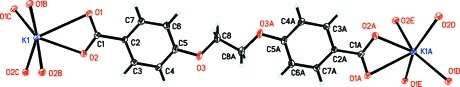
Coordination environment around the K ions in the title compound with the atom numbering scheme. Displacement ellipsoids are drawn at the 50% probability level. H atoms are presented as small spheres of arbitrary radius. [symmetry code: (A) -x-1, -y-1, -z; (B) -x, -y-1/2, -z+1/2; (C) -x, y+1/2, -z+1/2; (D) x-1, -y-3/2, z-1/2; (E) x-1, -y-1/2, z-1/2]

**Fig. 2.**
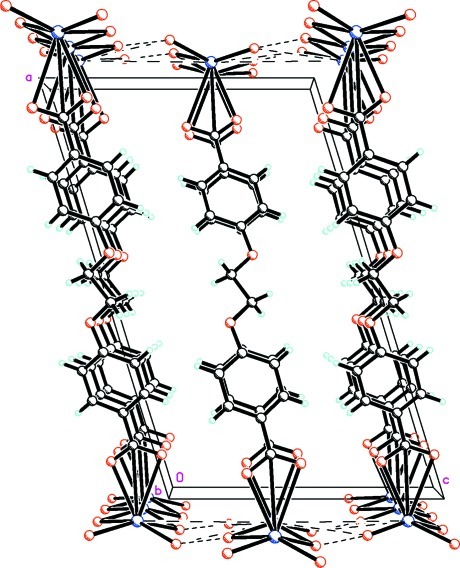
A view of the crystal packing along the b axis. Longer bonds between the K+ ions and O atoms are shown with dotted lines.

## supplementary crystallographic information

### Comment

The coordination chemistry of carboxylic compounds is attracting current attention, based on interesting properties like gas adsorption and separation, catalysis, magnetism, luminescence and host-guest chemistry (Su *et al.*, 2010; Zhu *et al.*, 2008) of these compounds. It is well-known that carboxylic acids are excellent building blocks for the construction of coordination polymers, yielding extended frameworks by virtue of their bridging abilities (Ma *et al.*, 2005; Zhang & Chen, 2008). Hence, the present paper aims to promote the search for new metal carboxylic complexes exhibiting special properties in many fields, in particular those bearing multicarboxylic-type ligands, a promising but still rather underdeveloped field of research. We report here the structure of a new polymeric dipotassium dicarboxylic compound (Fig. 1).

In the asymmetric unit one half of the anion is present. The anion lies on a crystallographic inversion center, which is located at the mid point of the C8—C8^i^ bond (symmetry code (i) = -*x*-1, -*y*-1, -*z*). All bond lengths and angles of the anion are within normal ranges (Allen *et al.*, 1987). The K^+^ ion is coordinated by six oxygen atoms, two from the ligand and four from neighboring ligands, in a distorted [KO_6_] polyhedron (Fig 2), whereby one anion coordinates all in all to ten K^+^ cations. The benzene rings of the anion are parallel to each other with a plane-to-plane distance of 3.488 Å. The carboxyl, aryl and O—CH_2_ moieties are coplanar with an r.m.s. deviation of 0.0638 Å.

A three-dimensional network is spanned owing to the coordination mode of the potassium cations. The K^+^ cations and the O atoms of the carboxylate anions form a layer parallel to (100). These layers are finally connected by the substituted ethane moieties into a three-dimensional structure (Fig. 2).

### Experimental

The precusor of the title compound was prepared by a reported procedure (Ma & Yang, 2011). The title compound was synthesized by the reaction of the precusor, diethyl 4,4'-(ethane-1,2-diyldioxy)-dibenzoate and potassium hydroxide in the conditions as follows: The precusor (1.0 g, 2.8 m*M*) and KOH (0.31 g, 5.6 m*M*) were put in water (150 cm^3^) in a 250 cm^3^ flask and the system was stirred for 24 h at 373 K for all solids dissolved and cooled down to room temperature. After filtration, a colorless solution was obtained. Evaporation of the solution gave a white solid (0.82 g, 77 %), which was washed with ethanol two times (10 ml each). Slow evaporation of a solution of the title compound in water led to the formation of colorless crystals, which were suitable for X-ray characterization.

### Refinement

Hydrogen atoms bonded to the C atoms of the anion were positioned geometrically and refined using a riding model with C—H = 0.93 - 0.97 Å and with Uiso(H) = 1.2 times Ueq(C). These hydrogen atoms were assigned isotropic thermal parameters and allowed to ride on their respective parent atoms.

### Crystal data

**Table:**

[K_2_(C_16_H_12_O_6_)]	*F*(000) = 388
*M**_r_* = 189.23	*D*_x_ = 1.614 Mg m^−^^3^
Monoclinic, *P*2_1_/*c*	Mo *K*α radiation, λ = 0.71073 Å
Hall symbol: -P 2ybc	Cell parameters from 7387 reflections
*a* = 18.0696 (7) Å	θ = 3.5–29.6°
*b* = 3.9866 (1) Å	µ = 0.64 mm^−^^1^
*c* = 11.3189 (5) Å	*T* = 298 K
β = 107.188 (2)°	Prism, colorless
*V* = 778.96 (5) Å^3^	0.15 × 0.11 × 0.10 mm
*Z* = 4	

### Data collection

**Table:**

Bruker SMART CCD area-detector diffractometer	2146 independent reflections
Radiation source: fine-focus sealed tube	1999 reflections with *I* > 2σ(*I*)
Graphite Monochromator monochromator	*R*_int_ = 0.018
phi and ω scans	θ_max_ = 29.6°, θ_min_ = 3.5°
Absorption correction: multi-scan (*SADABS*; Sheldrick, 1996)	*h* = −25→25
*T*_min_ = 0.919, *T*_max_ = 0.938	*k* = −5→4
7387 measured reflections	*l* = −15→15

### Refinement

**Table:**

Refinement on *F*^2^	Primary atom site location: structure-invariant direct methods
Least-squares matrix: full	Secondary atom site location: difference Fourier map
*R*[*F*^2^ > 2σ(*F*^2^)] = 0.023	Hydrogen site location: inferred from neighbouring sites
*wR*(*F*^2^) = 0.067	H-atom parameters constrained
*S* = 1.01	*w* = 1/[σ^2^(*F*_o_^2^) + (0.0361*P*)^2^ + 0.4108*P*] where *P* = (*F*_o_^2^ + 2*F*_c_^2^)/3
2146 reflections	(Δ/σ)_max_ < 0.001
109 parameters	Δρ_max_ = 0.39 e Å^−^^3^
0 restraints	Δρ_min_ = −0.22 e Å^−^^3^

### Special details

**Table:**

Geometry. All esds (except the esd in the dihedral angle between two l.s. planes) are estimated using the full covariance matrix. The cell esds are taken into account individually in the estimation of esds in distances, angles and torsion angles; correlations between esds in cell parameters are only used when they are defined by crystal symmetry. An approximate (isotropic) treatment of cell esds is used for estimating esds involving l.s. planes.
Refinement. Refinement of F^2^ against ALL reflections. The weighted R-factor wR and goodness of fit S are based on F^2^, conventional R-factors R are based on F, with F set to zero for negative F^2^. The threshold expression of F^2^ > 2sigma(F^2^) is used only for calculating R-factors(gt) etc. and is not relevant to the choice of reflections for refinement. R-factors based on F^2^ are statistically about twice as large as those based on F, and R- factors based on ALL data will be even larger.

### Fractional atomic coordinates and isotropic or equivalent isotropic displacement parameters (Å^2^)

**Table:**

	*x*	*y*	*z*	*U*_iso_*/*U*_eq_	
K1	−0.062276 (12)	0.69576 (6)	−0.14996 (2)	0.01343 (8)	
O1	0.10069 (4)	0.8108 (2)	0.01861 (7)	0.01551 (16)	
O2	0.08101 (4)	0.6705 (2)	−0.17911 (7)	0.01591 (16)	
O3	0.41949 (4)	1.2442 (2)	−0.07832 (8)	0.02053 (18)	
C1	0.12233 (6)	0.7855 (2)	−0.07712 (9)	0.01162 (18)	
C2	0.20252 (5)	0.9047 (3)	−0.07224 (9)	0.01196 (18)	
C3	0.23052 (6)	0.8522 (3)	−0.17345 (10)	0.01459 (19)	
H3A	0.1997	0.7410	−0.2428	0.018*	
C4	0.30339 (6)	0.9630 (3)	−0.17205 (10)	0.0162 (2)	
H4A	0.3215	0.9234	−0.2396	0.019*	
C5	0.34944 (6)	1.1339 (3)	−0.06932 (10)	0.0154 (2)	
C6	0.32378 (6)	1.1810 (3)	0.03393 (10)	0.0170 (2)	
H6A	0.3552	1.2870	0.1040	0.020*	
C7	0.25020 (6)	1.0670 (3)	0.03085 (9)	0.0151 (2)	
H7A	0.2327	1.1005	0.0994	0.018*	
C8	0.46737 (6)	1.4328 (3)	0.02248 (11)	0.0192 (2)	
H8A	0.4875	1.2908	0.0944	0.023*	
H8B	0.4383	1.6153	0.0442	0.023*	

### Atomic displacement parameters (Å^2^)

**Table:**

	*U*^11^	*U*^22^	*U*^33^	*U*^12^	*U*^13^	*U*^23^
K1	0.01384 (11)	0.01391 (12)	0.01382 (12)	−0.00019 (7)	0.00604 (8)	0.00031 (7)
O1	0.0156 (3)	0.0186 (4)	0.0148 (4)	−0.0019 (3)	0.0082 (3)	−0.0009 (3)
O2	0.0128 (3)	0.0207 (4)	0.0144 (3)	−0.0028 (3)	0.0041 (3)	−0.0033 (3)
O3	0.0129 (4)	0.0248 (4)	0.0266 (4)	−0.0065 (3)	0.0100 (3)	−0.0045 (3)
C1	0.0107 (4)	0.0106 (4)	0.0142 (4)	0.0004 (3)	0.0047 (3)	0.0010 (3)
C2	0.0101 (4)	0.0129 (4)	0.0132 (4)	−0.0003 (3)	0.0040 (3)	0.0014 (4)
C3	0.0126 (4)	0.0183 (5)	0.0132 (4)	−0.0010 (4)	0.0042 (4)	−0.0014 (4)
C4	0.0146 (4)	0.0207 (5)	0.0159 (5)	−0.0008 (4)	0.0083 (4)	0.0001 (4)
C5	0.0103 (4)	0.0155 (5)	0.0216 (5)	−0.0008 (3)	0.0066 (4)	0.0016 (4)
C6	0.0131 (4)	0.0203 (5)	0.0174 (5)	−0.0037 (4)	0.0042 (4)	−0.0041 (4)
C7	0.0135 (4)	0.0184 (5)	0.0148 (5)	−0.0020 (4)	0.0062 (4)	−0.0019 (4)
C8	0.0123 (4)	0.0186 (5)	0.0276 (6)	−0.0032 (4)	0.0073 (4)	−0.0011 (4)

### Geometric parameters (Å, º)

**Table:**

K1—O2^i^	2.6558 (8)	C1—C2	1.5104 (13)
K1—O1^ii^	2.6780 (8)	C2—C7	1.3886 (14)
K1—O2	2.7069 (8)	C2—C3	1.3981 (14)
K1—O1^iii^	2.7167 (8)	C3—C4	1.3845 (14)
K1—O2^iv^	2.8027 (8)	C3—H3A	0.9300
K1—O1	3.0335 (8)	C4—C5	1.3918 (15)
O1—C1	1.2599 (12)	C4—H4A	0.9300
O1—K1^ii^	2.6780 (8)	C5—C6	1.3914 (15)
O1—K1^iii^	2.7167 (8)	C6—C7	1.3957 (14)
O2—C1	1.2613 (12)	C6—H6A	0.9300
O2—K1^iv^	2.6558 (8)	C7—H7A	0.9300
O2—K1^i^	2.8027 (8)	C8—C8^v^	1.514 (2)
O3—C5	1.3719 (12)	C8—H8A	0.9700
O3—C8	1.4254 (14)	C8—H8B	0.9700
			
O2^i^—K1—O1^ii^	83.27 (2)	C2—C1—K1	163.16 (7)
O2^i^—K1—O2	81.85 (2)	O1—C1—K1^i^	130.55 (7)
O1^ii^—K1—O2	120.61 (2)	O2—C1—K1^i^	52.82 (5)
O2^i^—K1—O1^iii^	158.48 (3)	C2—C1—K1^i^	85.79 (5)
O1^ii^—K1—O1^iii^	95.29 (2)	K1—C1—K1^i^	77.91 (2)
O2—K1—O1^iii^	116.57 (2)	C7—C2—C3	118.26 (9)
O2^i^—K1—O2^iv^	93.79 (2)	C7—C2—C1	121.84 (9)
O1^ii^—K1—O2^iv^	159.03 (2)	C3—C2—C1	119.90 (9)
O2—K1—O2^iv^	79.21 (2)	C4—C3—C2	121.06 (10)
O1^iii^—K1—O2^iv^	79.87 (2)	C4—C3—K1^i^	124.14 (7)
O2^i^—K1—O1	103.98 (2)	C2—C3—K1^i^	86.80 (6)
O1^ii^—K1—O1	84.40 (2)	C4—C3—H3A	119.5
O2—K1—O1	45.30 (2)	C2—C3—H3A	119.5
O1^iii^—K1—O1	97.22 (2)	K1^i^—C3—H3A	59.1
O2^iv^—K1—O1	116.36 (2)	C3—C4—C5	119.85 (9)
C1—O1—K1^ii^	137.18 (7)	C3—C4—H4A	120.1
C1—O1—K1^iii^	127.26 (6)	C5—C4—H4A	120.1
K1^ii^—O1—K1^iii^	95.29 (2)	O3—C5—C6	124.26 (10)
C1—O1—K1	86.40 (6)	O3—C5—C4	115.58 (9)
K1^ii^—O1—K1	95.60 (2)	C6—C5—C4	120.15 (9)
K1^iii^—O1—K1	82.78 (2)	C5—C6—C7	119.12 (10)
C1—O2—K1^iv^	147.07 (7)	C5—C6—H6A	120.4
C1—O2—K1	101.75 (6)	C7—C6—H6A	120.4
K1^iv^—O2—K1	101.23 (3)	C2—C7—C6	121.51 (9)
C1—O2—K1^i^	106.17 (6)	C2—C7—H7A	119.2
K1^iv^—O2—K1^i^	93.79 (2)	C6—C7—H7A	119.2
K1—O2—K1^i^	97.56 (3)	O3—C8—C8^v^	105.46 (12)
C5—O3—C8	117.68 (9)	O3—C8—H8A	110.6
O1—C1—O2	124.48 (9)	C8^v^—C8—H8A	110.6
O1—C1—C2	118.78 (9)	O3—C8—H8B	110.6
O2—C1—C2	116.73 (9)	C8^v^—C8—H8B	110.6
O1—C1—K1	70.54 (5)	H8A—C8—H8B	108.8
O2—C1—K1	55.63 (5)		
			
O2^i^—K1—O1—C1	−55.46 (6)	K1—O2—C1—K1^i^	−101.55 (4)
O1^ii^—K1—O1—C1	−137.08 (7)	O2^i^—K1—C1—O1	126.93 (6)
O2—K1—O1—C1	7.68 (5)	O1^ii^—K1—C1—O1	43.72 (7)
O1^iii^—K1—O1—C1	128.28 (6)	O2—K1—C1—O1	−165.70 (10)
O2^iv^—K1—O1—C1	46.04 (6)	O1^iii^—K1—C1—O1	−56.46 (7)
O2^i^—K1—O1—K1^ii^	81.62 (3)	O2^iv^—K1—C1—O1	−139.16 (6)
O1^ii^—K1—O1—K1^ii^	0.0	C1^iv^—K1—C1—O1	−158.75 (5)
O2—K1—O1—K1^ii^	144.76 (4)	C3^iv^—K1—C1—O1	−147.22 (6)
O1^iii^—K1—O1—K1^ii^	−94.64 (2)	K1^iii^—K1—C1—O1	−39.24 (5)
O2^iv^—K1—O1—K1^ii^	−176.88 (2)	K1^vi^—K1—C1—O1	−97.19 (6)
O2^i^—K1—O1—K1^iii^	176.26 (2)	K1^vii^—K1—C1—O1	82.81 (6)
O1^ii^—K1—O1—K1^iii^	94.64 (2)	O2^i^—K1—C1—O2	−67.37 (5)
O2—K1—O1—K1^iii^	−120.60 (4)	O1^ii^—K1—C1—O2	−150.59 (6)
O1^iii^—K1—O1—K1^iii^	0.0	O1^iii^—K1—C1—O2	109.24 (6)
O2^iv^—K1—O1—K1^iii^	−82.24 (3)	O2^iv^—K1—C1—O2	26.54 (6)
O2^i^—K1—O2—C1	111.19 (5)	O1—K1—C1—O2	165.70 (10)
O1^ii^—K1—O2—C1	34.03 (7)	C1^iv^—K1—C1—O2	6.95 (7)
O1^iii^—K1—O2—C1	−80.53 (7)	O2^i^—K1—C1—C2	0.4 (2)
O2^iv^—K1—O2—C1	−153.35 (6)	O1^ii^—K1—C1—C2	−82.8 (2)
O1—K1—O2—C1	−7.82 (6)	O2—K1—C1—C2	67.7 (2)
O2^i^—K1—O2—K1^iv^	−92.56 (4)	O1^iii^—K1—C1—C2	177.0 (2)
O1^ii^—K1—O2—K1^iv^	−169.72 (2)	O2^iv^—K1—C1—C2	94.3 (2)
O1^iii^—K1—O2—K1^iv^	75.72 (3)	O1—K1—C1—C2	−126.6 (3)
O2^iv^—K1—O2—K1^iv^	2.91 (2)	O2^i^—K1—C1—K1^i^	−14.40 (2)
O1—K1—O2—K1^iv^	148.43 (4)	O1^ii^—K1—C1—K1^i^	−97.62 (2)
O2^i^—K1—O2—K1^i^	2.85 (2)	O2—K1—C1—K1^i^	52.97 (6)
O1^ii^—K1—O2—K1^i^	−74.31 (3)	O1^iii^—K1—C1—K1^i^	162.21 (2)
O1^iii^—K1—O2—K1^i^	171.14 (2)	O2^iv^—K1—C1—K1^i^	79.51 (2)
O2^iv^—K1—O2—K1^i^	98.32 (4)	O1—K1—C1—K1^i^	−141.33 (6)
O1—K1—O2—K1^i^	−116.16 (4)	O1—C1—C2—C7	−5.67 (15)
K1^ii^—O1—C1—O2	−108.67 (11)	O2—C1—C2—C7	173.34 (10)
K1^iii^—O1—C1—O2	63.78 (13)	O1—C1—C2—C3	174.29 (9)
K1—O1—C1—O2	−14.32 (10)	O2—C1—C2—C3	−6.70 (14)
K1^ii^—O1—C1—C2	70.25 (13)	C7—C2—C3—C4	−0.94 (16)
K1^iii^—O1—C1—C2	−117.29 (9)	C1—C2—C3—C4	179.10 (10)
K1—O1—C1—C2	164.60 (8)	C7—C2—C3—K1^i^	−129.43 (9)
K1^ii^—O1—C1—K1	−94.35 (8)	C1—C2—C3—K1^i^	50.60 (9)
K1^iii^—O1—C1—K1	78.10 (6)	C2—C3—C4—C5	−0.93 (17)
K1^ii^—O1—C1—K1^i^	−40.84 (13)	K1^i^—C3—C4—C5	108.31 (10)
K1^iii^—O1—C1—K1^i^	131.62 (6)	C8—O3—C5—C6	−3.09 (16)
K1—O1—C1—K1^i^	53.52 (7)	C8—O3—C5—C4	177.44 (10)
K1^iv^—O2—C1—O1	−116.98 (12)	C3—C4—C5—O3	−177.77 (10)
K1—O2—C1—O1	16.41 (11)	C3—C4—C5—C6	2.74 (17)
K1^i^—O2—C1—O1	117.97 (9)	O3—C5—C6—C7	177.93 (10)
K1^iv^—O2—C1—C2	64.08 (15)	C4—C5—C6—C7	−2.63 (17)
K1—O2—C1—C2	−162.53 (7)	C3—C2—C7—C6	1.04 (16)
K1^i^—O2—C1—C2	−60.98 (9)	C1—C2—C7—C6	−179.00 (10)
K1^iv^—O2—C1—K1	−133.39 (13)	C5—C6—C7—C2	0.73 (17)
K1^i^—O2—C1—K1	101.55 (4)	C5—O3—C8—C8^v^	−170.63 (11)
K1^iv^—O2—C1—K1^i^	125.05 (13)		

Symmetry codes: (i) −*x*, *y*+1/2, −*z*−1/2; (ii) −*x*, −*y*+2, −*z*; (iii) −*x*, −*y*+1, −*z*; (iv) −*x*, *y*−1/2, −*z*−1/2; (v) −*x*+1, −*y*+3, −*z*; (vi) *x*, *y*−1, *z*; (vii) *x*, *y*+1, *z*.
